# Romanian Dendrocoelidae Hallez, 1892 (Platyhelminthes, Tricladida, Dendrocoelidae) Revisited: A Tribute to Radu Codreanu and Doina Balcesco

**DOI:** 10.3390/biology14070887

**Published:** 2025-07-19

**Authors:** Anda Felicia Babalean

**Affiliations:** Department of Biology and Environmental Engineering, Faculty of Horticulture, University of Craiova, 13 A. I. Cuza Street, 200396 Craiova, Romania; anda.babalean@ucv.ro

**Keywords:** dendrocoelidae, morphological characters, palaeogeographical conditions, natural history

## Abstract

This paper presents the current state of knowledge on the Romanian Dendrocoelidae as part of the European/Palearctic Dendrocoelidae, emphasizing the contributions of the Romanian zoologists Radu Codreanu and Doina Balcesco. The main objective of this work was to identify the knowledge gaps for future alignment with current standards. This article presents the species inventory and a short historical overview of the classical phylogenetic system and discusses some morphological characters used in the systematics of the group. This study also analyzes the arguments (and hypotheses) put forward by Codreanu, Balcesco, and other authors regarding the phylogenetic value of various factors, including (a) the position of the oviducts between the male atrium and the bursal canal (typical for *Paradendrocoelum*); (b) the eyes and the penial flagellum in relation to the palaeogeographical context governed by the Quaternary Glaciation; and (c) the point of view of Codreanu and Balcesco on the origin and composition of the actual Romanian Dendrocoelidae fauna. The major key finding is that the Dendrocoelidae species in Romania should be reinvestigated in an integrative way, and specific research needs and future directions are suggested.

## Simple Summary

The European Dendrocoelidae are an important group of freshwater flatworms. From the first species described by Müller in 1774, *Dendrocoelum lacteum*, the current account comprises nearly 100 species; many of them are endemic, with a geographical range restricted to a small area, and blind, inhabiting dark environments. The species were historically included in a system of nine units (taxa) that were difficult to distinguish clearly based on their morphology, as one or more character were often shared among units. The classical literature operates with the taxa *Polycladodes*, *Dendrocoelum*, *Dendrocoelides*, *Paradendrocoelum*, *Palaeodendrocoelum*, *Eudendrocoelum*, *Neodendrocoelum*, *Bolbodendrocoelum*, and *Apodendrocoelum*. The endemicity and the diversity of the morphological characters make it difficult to achieve a clear understanding of the taxonomic diversity and natural history of the group. How many species are there? What is their history? When did they evolve? To what extent are they related? The Dendrocoelidae of Romania are part of Europe’s freshwater diversity. A major knowledge gap is the lack of type specimens, the specimens upon which the species description was made. The Romanian Dendrocoelidae are a missing piece of a blurred, unresolved puzzle. These questions can be addressed by combining classical morphology with modern genetic tools.

## 1. Introduction

The Dendrocoelidae Hallez, 1892, is an important family of triclad freshwater worms belonging to the suborder Continenticola [1]. The main characteristic of this family is the alternative disposition of the longitudinal and circular muscular layers in the pharynx [1,2]. The family Dendrocoelidae consists of 22 genera with a large distribution, out of which only *Dendrocoelum* and *Polycladodes* are present in the Romanian fauna [1,3]. The knowledge of European Dendrocoelidae began with the description of *Dendrocoelum lacteum* by Müller in 1774. A survey of the available literature [2,3,4,5,6,7,8,9,10,11] increased the current count to 100 Palearctic species. Once described, the species were included in a system of supraspecific taxa, either at the rank of genus or subgenus: *Dendrocoelum*, *Dendrocoelides*, *Eudendrocoelum*, *Neodendrocoelum*, *Bolbodendrocoelum*, *Paradendrocoelum*, *Apodendrocoelum*, *Palaeodendrocoelum*, and *Polycladodes* (see paragraph 3). The most recent literature recognizes only the genera *Dendrocoelum* and *Polycladodes* [1,11], with *Dendrocoelum* s.l. representing the consolidation of the remaining taxa. In this paper, I use the term Dendrocoelidae for the only two genera present in the Romanian fauna, *Dendrocoelum* and *Polycladodes*.

The Dendrocoelidae of Romania were studied by several scientists, especially from a faunal and systematic point of view. The faunal approach was utilized by de Beauchamp, del Papa, Codreanu, Codreanu and Balcesco, and Stocchino and coauthors [3,12,13,14,15,16,17,18,19,20,21], who identified and described new species. The systematics of Palearctic Dendrocoelidae have been a matter of prolonged debate, with revisions by many zoologists, including Komárek, Stanković and Komárek, Kenk, de Beauchamp, and Reisinger and Gourbault [5]. Despite all the morphologically based revisions, the phylogenetic systematics remain unresolved; however, it is expected to be continuously reshaped by modern tools, as anticipated in other studies [22]. Significant research efforts using modern tools (barcoding, multilocus phylogenetic, genomic analyses, etc.) and combined molecular and morphological analyses have explored and unraveled many conundrums related to the taxonomic biodiversity, evolution, evolutionary dynamics, speciation patterns, systematics, and phylogeny of Tricladida at both lower and higher levels [11,22,23,24,25,26,27,28,29,30,31,32,33,34,35,36,37].

This paper offers an overview of the Romanian Dendrocoelidae as part of the European/Palearctic group, emphasizing the historical contributions of Radu Codreanu and Doina Balcesco. Their efforts to study this group of worms are part of the broader historical endeavor to learn about the entire European/Palearctic group. Therefore, even though only the genera *Dendrocoelum* and *Polycladodes* are currently recognized, the analysis and discussions of the former *Dendrocoelum* s.l. divisions are considered essential for this paper.

The term “morphological character” refers to a part of the body (head, pharynx, eye, etc.), while the term “morphological characteristic” refers to a property, an attribute of a character (white, overdeveloped, etc.).

## 2. Methodology

The literature review was performed using various databases: Romanian libraries, MNHN library (Muséum National d Histoire Naturelle Paris), Google and Google Scholar, ResearchGate, BHL (Biodiversity Heritage Library), EurekaMag, NHBS (Natural History Book Service), etc. Keywords related to this article were used: *Dendrocoelidae*, *Dendrocoelum species*, systematic, planarian molecular phylogeny, etc. The final reference list of all accessed articles served as the starting point for new searches using the article and journal titles. Some articles were kindly provided to me by specialists and colleagues in the field.

## 3. Species Inventory

The species inventory, shown in Table 1, follows, in part, the system of Sluys et al. for the genera *Dendrocoelum* and *Polycladodes*, and Gourbault for the *Dendrocoelum* s.l. subgenera [1,2].

The deposition of the type specimens of the species authored by Codreanu and Balcesco is not recorded in any publications, suggesting that they may be lost or are private property; thus, this material is unavailable for further comparative studies. The search for the histological slides would require significant effort. This should be the subject of a separate study that may reveal the type specimens or the need for a recollection strategy and protocols for neotype designation.

## 4. The Classical Phylogenetic System, Morphological Types, and Taxa

With the increase in the number of species, the systematics of Dendrocoelidae has become complicated, undergoing several changes according to the point of view of different authors. Historically, species have been grouped into several morphological types defined by sets of morphological characters, with each morphological type corresponding to a supraspecific taxon at the genus or subgenus rank. The morphologically established taxa were as follows: *Dendrocoelum* (s.l. and s.str.), *Polycladodes*, *Dendrocoelides*, *Eudendrocoelum*, *Neodendrocoelum*, *Paradendrocoelum*, *Bolbodendrocoelum*, *Apodendrocoelum*, and *Palaeodendrocoelum*. The separation between them can be better understood from a historical perspective, as presented by Gourbault [2].

*Dendrocoelum*

**Figure 1:**
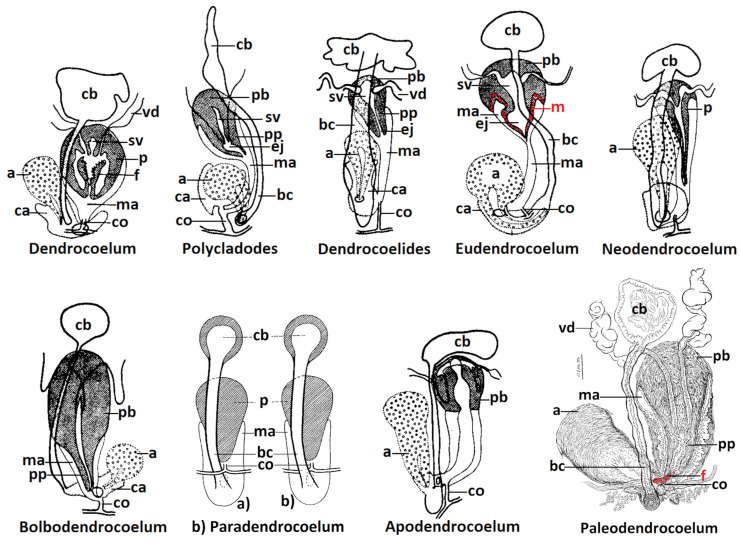
The morphological types corresponding to the supraspecific taxa *Dendrocoelum* s.str., *Polycladodes*, *Dendrocoelides*, *Eudendrocoelum*, *Neodendrocoelum*, *Bolbodendrocoelum*, *Apodendrocoelum* (adapted from Gourbault 1972 [2] (p. 43, Figure 7)), *Paradendrocoelum* (adapted from Kenk 1930 [38] (p. 56, Figure 2)), and *Palaeodendrocoelum* (adapted from Codreanu 1950 [17] (p. 611, Figure 3, scale bar 0.1 mm). Abbreviations: a—adenodactyl; bc—bursal canal; ca—common atrium; cb—copulatory bursa; co—common oviduct; ej—ejaculatory duct; f—flagellum; m—supplementary muscular circular layer at the base of the penis bulb; ma—male atrium; p—penis; pb—penis bulb; pp—penis papilla; sv—seminal vesicle; vd—vas deferens.

*Dendrocoelum* was created at the genus rank by Oersted, 1844, for the species *lacteum,* which is the type species. The systematics of Gourbault recognizes the genus *Dendrocoelum* (*Dendrocoelum* s.l.), which is separated into eight subgenera, including the subgenus *Dendrocoelum* (*Dendrocoelum* s. str.) with two species: the occulated *D. lacteum* and the anophthalmic *D. infernale*. [2]. The distinctive characteristic of the subgenus *Dendrocoelum* (*Dendrocoelum* s. str.) is the presence of a true flagellum, which is defined as a long structure with longitudinal muscles covered by a vacuolated epithelium, derived from and attached to the inner epithelium of the penial papilla; it can be invaginated (inverted) or everted out of the seminal vesicle [5,9]. Other characteristics include a globular penis with a large seminal vesicle, a short male atrium, the communication between the male atrium and the common atrium through an orifice, and the opening of the common oviduct into the male atrium (Figure 1).

*Polycladodes*

*Polycladodes* was created by Steinmann in 1910, at the genus level. for the pluri-occulated species *Polycladodes alba* (type species). The distinctive character of *Polycladodes* is a supplementary longitudinal muscular layer in the external area of the pharynx, a character identifying the current genus rank [1]. Other morphological characters extracted from Gourbault for *Polycladodes* are the lack of a flagellum, a long and tubular male atrium, the communication between the male atrium and the common atrium through a short duct with no sphincter, and the opening of the common oviduct into a short area at the meeting place of the male atrium and the common atrium [2] (Figure 1). Gourbault included four species in *Polycladodes*: the pluri-occulated *P. album* with 15–30 eyes and the anophthalmic *P. caecum*, *P. voinovi*, and *P. affine* [2].

*Dendrocoelides*

This taxon was created by de Beauchamp in 1919, at the genus level, for the species *Dendrocoelides regnardi*. The distinctive characteristic of *Dendrocoelides* is the lack of a flagellum, in contrast to *Dendrocoelum* s.str. (Figure 1).

Gourbault considered *Dendrocoelides* at the subgenus rank and included 28 species in it, out of which only two are occulated: *Dl. lescherae* in France and *Dl. vaillanti* in Algeria. Four species in this group (*Dl. lescherae, Dl. mrazeki panonicum, Dl. stenophalus,* and *Dl. racovitzai*) possess an invaginable penis papilla, in other words, a flagelliform papilla, thus showing affinity with *Dendrocoelum* [2].

*Eudendrocoelum*

*Eudendrocoelum* was created by Komarek in 1926, at the genus level, to include three species: *lacteum, infernale,* and *subterraneum*. The distinctive characteristic of *Eudendrocoelum* is the presence of a supplementary muscular circular layer starting at the base of the penis bulb, which thins along the wall of the penis papilla (Figure 1). In 1932, de Beauchamp separated the species *lacteum* and *infernale* into the genus *Dendrocoelum*. The movement of these two species from one taxon to another shows a clear intersection of *Eudendrocoelum* with *Dendrocoelum***.** Gourbault, with respect to the flagellum, included a mixture of 10 species in *Eudendrocoelum*: (i) flagellum absent in some species, showing affinity with *Dendrocoelides,* and (ii) a flagelliform structure, invaginated or not, present in some species, with *E. sollaudi, remyi, gineti,* and *tubuliferum* showing affinity with *Dendrocoelum*. Most species are unpigmented and anophthalmic. Only one species, *D. (E.) parvioculatum,* has both eyes and a demi-invaginated penis papilla [2].

*Neodendrocoelum*

This taxon was created by Komárek in 1926. The diagnosis given by Gourbault (1972) for *Neodendrocoelum* at the subgenus rank includes the great development of a penis and adenodactyl (Figure 1), the histology of the oviducts, and the glands of the genital pore. Gourbault included 13 occulated species in *Neodendrocoelum*; most of them are pigmented with a species-specific dorsal pattern and distributed mainly in former Yugoslavia, in Lake Ohrid and tributaries [2]. This taxon is absent in Romania.

*Bolbodendrocoelum*

This taxon was created by de Beauchamp in 1932, at the subgenus level, for the anophthalmic species *agile* [2]. *Bolbodendrocoelu* is monospecific, and its distinctive character is an overdeveloped penis bulb (Figure 1). It is absent in Romania.

*Paradendrocoelum*

This subgenus was created by Kenk in 1930 for the species *cavaticum,* based on the position of the oviducts between the male atrium and the bursal canal (Figure 1). Six hypogeic, anophthalmic species—*cavaticum*, *spelaeum*, *infernale*, *tubuliferum*, *hankói,* and *carpathicum*—were assigned by Kenk to *Paradendrocoelum* [38]. One Romanian species—*P. alexandrinae*—was also assigned by Codreanu and Balcesco to *Paradendrocoelum* [21].

*Apodendrocoelum*

*Apodendrocoelum* was created by de Beauchamp in 1932 at the subgenus rank. Its distinctive characteristic is a greatly reduced penis papilla (Figure 1). *Apodendrocoelum* contains four anophthalmic species: *A. brachyphallus* and *A. lipophallus* in Romania, and *A. puteale* and one species with insufficient diagnosis in Germany [2].

*Palaeodendrocoelum*

This genus was created by Codreanu in 1949–1950 based on the external morphology (pigmentation, eyes, and adhesive organ) combined with genital characteristics. The diagnosis given by Codreanu [17] included the following characteristics: small size (9 mm × 1 mm, specific pigmentation, numerous eyes (14–31) with a particular disposition, differentiated and infra-nucleated adhesive organ, a non-invaginated flagellum, male and common atrium completely separated, and the position of the oviducts between the male atrium and the bursal canal showing affinity with *Paradendrocoelum* (Figure 1).

A group of interest is represented by the species found in Lake Ohrid. Kenk presented a group of 17 occulated *Dendrocoelum* species, authored by Müller, Stanković, Stanković and Komárek, and Kenk [5]. Of these species, only two bear a true flagellum (*D. lacteum, D. cruciferum*); most of the others have various types of penial papilla: inversible, invertible, pseudoflagellum, etc. [5]. Kenk showed the penis papilla variability, which he attributed to the physiological state, fixatives, or even intrapopulational variability. Some of these species have a circular muscular layer at the base of the penis bulb (the wall of the penis papilla) [5], showing affinity with *Eudendrocoelum*. Nearly 10 of these 17 species were considered as belonging to *Neodendrocoelum* by Gourbault, including *D. cruciferum*, a species with a true flagellum [2].

## 5. The Morphological Characters of Dendrocoelidae and Their Phylogenetic Value

The characters used in both the older and more recent literature (for species diagnosis and systematics) include both the external and internal morphology.

The external morphology includes body pigmentation, eyes, and adhesive organs. The internal morphology includes a multitude of characters, with some of them already having been presented: pharynx musculature; position of the oviducts; the dorsal/ventral position of the testes; the communication of the vas deferens with the penis and the adenodactyl; the characteristics of the copulatory apparatus (such as the location) and the penis bulb (the arrangement of the musculature and the degree of development); the shape and size of the seminal vesicle; the type of flagellum when present; the degree and way of separation of the male and common atria; the bursal canal; the size ratio of penis to adenodactyl; and the development and disposition of the eosinophilic glands, etc.

The phylogenetic value of the morphological characteristics is especially evident when they are correlated with other aspects, such as the paleogeographic conditions and the geological ‘‘moment’’ of appearance, that is, their age. For systematic phylogeneticists, this is complex work, requiring the establishment of the synapomorphies and autapomorphies [39,40].

The taxonomic value of some characteristics is as follows:

(1) The position of the oviducts between the male atrium and the bursal canal was used to establish the taxon *Paradendrocoelum*.

The taxonomic value of this characteristic is seen differently by different zoologists.

(A) As an invalid generic characteristic.

Gourbault considered that the genus *Paradendrocoelum* cannot be preserved because Codreanu showed evidence of the variability of this characteristic “dans une même espèce” [2], but no information about the species, journal, and year of publication was given.

De Beauchamp considered the characteristic as a secondary one later in Dendrocoelidae evolution; thus, it is only a specific characteristic and not a generic one [17].

(B) As a valid generic characteristic.

**Figure 2:**
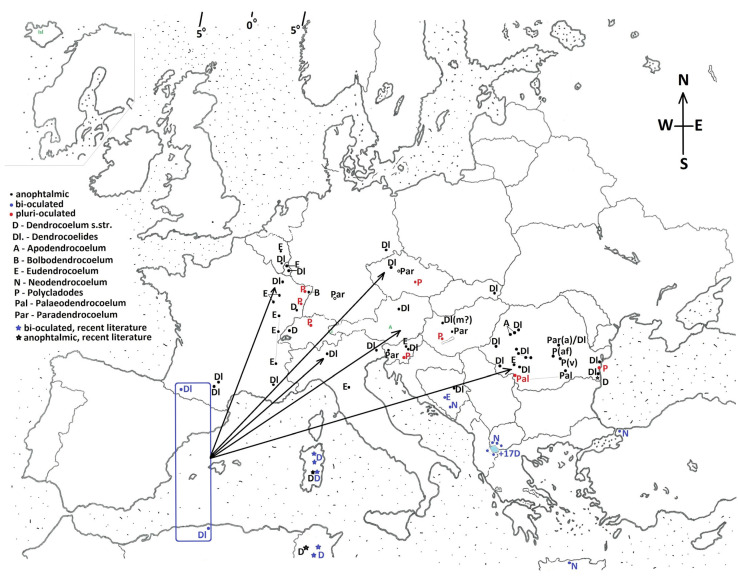
The geographical distribution of the actual Dendrocoelidae and the biogeographical (paleo-geographical) context supporting the hypothesis of *Dendrocoelides’* evolution and diversification, determined by the Quaternary Glaciation.

For two reasons, Codreanu considered this characteristic to be valuable:

(a) This characteristic is present in a group of hypogeic European species, including *cavaticum sollaudi, hankoi, spelaeum,* and one unnamed sp. (affinities between species), in opposition to the group *sphaerophalus, carpathicum, tubuliferum,* and *infernale*, species considered to be of evidently different origins [17].

(b) As this characteristic is present in all the Holarctic genera, *Dendrocoelopsis*, *Amyadenium*, *Miodendrocoelum,* etc., it is considered a primitive (primary) characteristic, one of the first in the evolution of Dendrocoelidae [17].

(2) The taxonomic value of the eyes and the biogeographical context (Figure 2 Map).

Hypotheses and arguments.

(A) Kenk and de Beauchamp considered that the eyes have no generic value; they disappear in the hypogeic species, which are closely related. The lack of eyes is seen as a regressive adaptive convergence in relation to subterranean life [17].

Examples of related occulated and anophthalmic species belonging to the same genus are given to support this theory: the pluri-occulated *Polycladodes alba* and the anophthalmic *Polycladodes affine* [17] and *Polycladodes voinovi*; the occulated *Dendrocoelum lacteum* and the anophthalmic *Dendrocoelum infernale* [17]; and the pluri-occulated *Palaeodendrocoelum romanodanubialis* and the anophthalmic *Palaeodendrocoelum getticum*.

(B) A second group of theories assigns generic value to eyes. Species of Tertiary origin, relics in the actual fauna, are occulated, while the anophthalmic *Dendrocoelides* are the result of the Quaternary Glaciation. These theories are supported by Beclemișev, Stanković, and Codreanu [17].

(a) The severe Glaciation in North and Central Europe was the driving force for speciation. Most epigeic Dendrocoelidae disappeared. Some species underwent a subterranean migration followed by the adaptive loss of eyes. This is the case for the Romanian *Dendrocoelides* anophthalmic species. After migration into the subterranean and adaptive eye loss, a process of diversification followed at the level of the copulatory apparatus (evolutive divergences), giving numerous anophthalmic endemic species, which are distinct at the level of genitalia [18].

(b) Attenuated Glaciation in S Europe determined the survival of some species (Tertiary species) in lakes, springs, and rivers as Tertiary relics. *Palaeodendrocoelum romanodanubiale* is a relic. *Palaeodendrocoelum* has the genus rank [17].

The age/oldness of the morphological characters in geological time is essential in tracing phylogenies. Regarding the eyes and copulatory apparatus, one question arises: which is the primary character (the primitive character), the eyes or the copulatory apparatus? This question generated the following two hypotheses:

Eyes first?

Codreanu considered the eyes as the primitive character [17], while the lack of eyes means the loss of this character. In this situation, the genus/subgenus *Dendrocoelum* s.str. should be considered the starting point in evolution. Such an evolutionary pattern involves the loss of the penial flagellum (*Dendrocoelum* s.str. has a more complex copulatory apparatus due to the presence of the flagellum).

Copulatory apparatus first?

Gourbault considered only the penis to be important in establishing the systematic position [2]. The subgenus *Dendrocoelides* (in which most species are anophthalmic), having the simplest penis, represents the starting point in evolution, while the subgenus *Dendrocoelum* s.str. has the highest evolutionary position due to the penial flagellum, which is considered an evolutionary acquisition.

## 6. Discussion

The literature review reveals various knowledge gaps.

The Dendrocoelidae fauna of Romania consists of 21 species. Of these species, one has a wide European distribution, *Polycladodes album*, with the rest being endemic. More than half of the species (thirteen species) were authored by Codreanu, and Codreanu and Balcesco [15,16,17,18,19,20,21], species for which the deposition of the type specimens is unknown. The diagnosis of some species is incomplete, with no figurative reconstruction of the copulatory apparatus, including *Dendrocoelum (Polycladodes) affine*, *Dendrocoelum (Paradendrocoelum) alexandrinae,* and *Dendrocoelum (Palaeodendrocoelum*) *getticum* [21]. These deficiencies impair the knowledge of the whole group.

Some of the morphological types presented are very distinct and unmistakable, such as *Bolbodendrocoelum* and *Apodendrocoelum,* making a clear diagnosis of the taxa possible. For the rest of the taxa (regardless of the genus or subgenus rank), a clear diagnosis is not possible, as one or more characters are shared across multiple morphological types/taxa. Many supraspecific taxa include a mix of species with a combination of morphological character, making them very difficult to order. This could be a sound reason to abandon these taxonomic divisions, at least temporarily. Some species may not be accurately defined, some may not be good species and might fall under synonymy, and some others may not belong to the genus they were originally attributed. The taxa *Paradendrocoelum* and *Palaeodendrocoelum* seem to be the most problematic.

**Figure 3:**
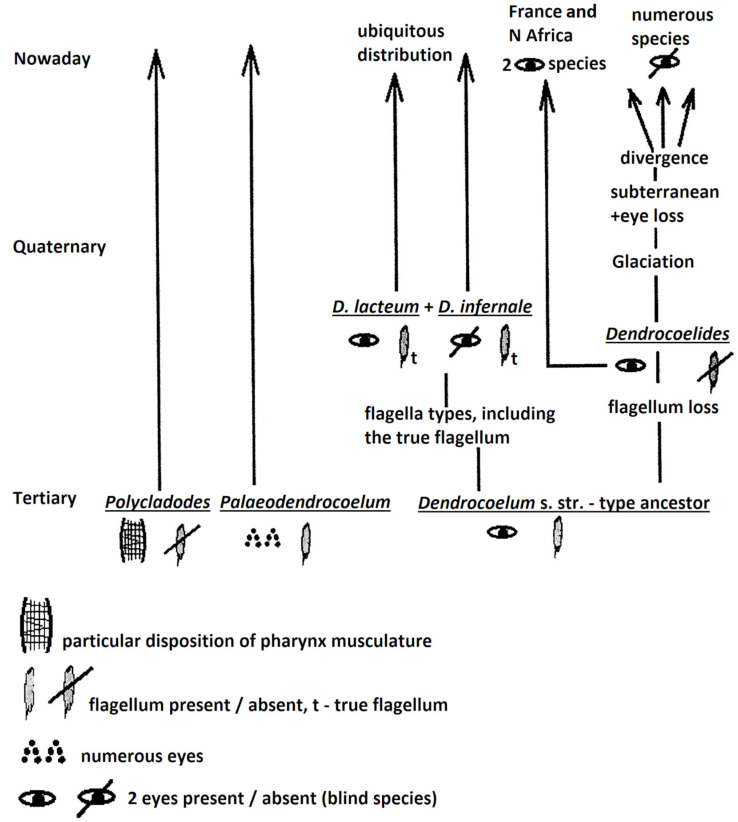
An evolutionary pattern with a *Dendrocoelum*-type ancestor, according to the author’s judgment.

**Figure 4:**
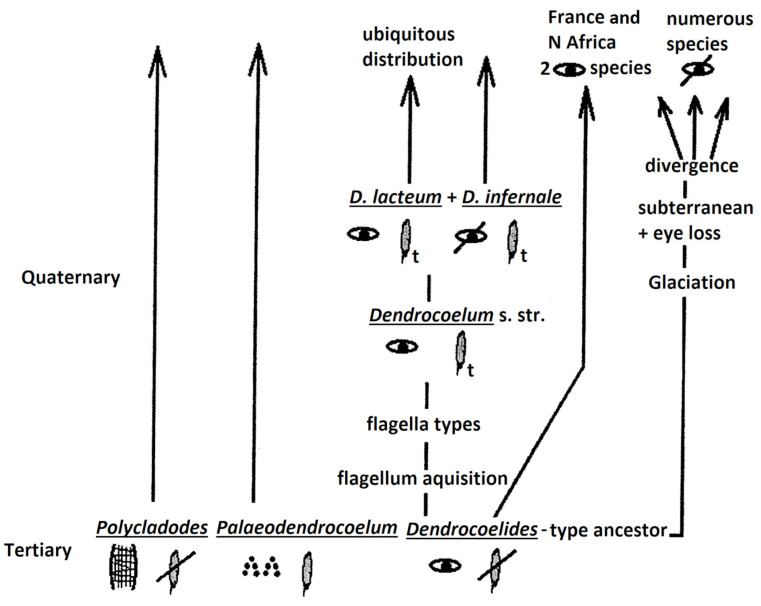
An evolutionary pattern with a *Dendrocoelides*-type ancestor, according to the author’s judgment.

Using the eyes and the penial flagellum, and based on the hypotheses put forward by all the authors cited in this paper, I suggest two main evolutionary patterns:

(1) One evolutionary pattern (Figure 3) may consider three groups (taxa) of ancient origin: *Polycladodes* and *Paleodendrocoelum* (of Tertiary origin), and a *Dendrocoelum*-type ancestor (of unknown geological origin), which is bi-occulated and has a penial flagellum of an unknown type. The evolution of the latter *Dendrocoelum*-type ancestor may have followed two main directions:

(1) (a) Flagellum preservation and its evolution in the various flagellar types found in the current fauna.

(1) (b) A loss of the flagellum, a characteristic typical of *Dendrocoelides*. This type of ancestor (of pre-Quaternary age) underwent Glaciation (see pages 8–9 of this paper, point (2) (B)). Attenuated Glaciation in some areas (S Europe and N Africa) determined the survival of two ocellated species in France and N Africa, which represent a margin of an ancient geographical range. Other *Dendrocoelides* species disappeared, and others migrated into the subterranean, where they lost their eyes and underwent diversification, resulting in numerous endemic species that are distinct at the level of genitalia.

(2) A second model (Figure 4) may also consider three groups (taxa) of ancient origin, out of which *Polycladodes* and *Paleodendrocoelum* are of Tertiary origin. The third group may be of the *Dendrocoelides* type, occulated and without penial flagellum, following two evolutionary paths:

(2) (a) Flagellum acquisition, giving various types of flagella.

(2) (b) Evolution induced by the Glaciation, with the preservation of the two ocellated species in the current fauna (in France and N Africa), also with subterranean radiation.

Both patterns suggest the following conclusions: a lack of eyes means they were lost, and the penial flagellum may have multiple independent origins: *Palaeodendrocoelum* and the *Dendrocoelum*-type via a *Dendrocoelides* ancestor (Figure 4). Amongst invertebrates, molecular data have indicated independent evolution (dual origin) in even more unexpected cases, such as the dual origin of striated musculature [41].

Looking at the map of the current distribution of *Dendrocoelides* (Dl)—Figure 2—the most likely evolutionary model appears to be the one shown in Figure 4. It is very probable that the taxon *Dendrocoelides* has an ancient origin, before the European and North African plates separated. Thus, the *Dendrocoelides* represent the starting point in evolution.

Regarding the flagellar types, synthesized by Stocchino and coauthors [9], some observations can be made: The histological structure of the penial flagellum is not known in all species. Most species (thirteen) possess a pseudoflagellum; of these, ten species are occulated and found in Lake Ohrid. Two species out of the four possessing a completely inverted penial papilla are occulated and are found in Lake Ohrid. The phylogenetic value of the penial papilla types (the flagellar types) should address the following issues: the variability of the penis shape indicated by Kenk [5] (individual variability and variability in living and fixed worms), and whether the histological structure of the flagellum reflects a physiological state (for instance, the vacuolated epithelium).

The natural history of this group is very difficult to reconstruct on morphological grounds alone. Numerous morphological types may have been lost over geological time, no longer present in the actual fauna or preserved in fossils. Additionally, it is completely unknown where and how many transitions from epigeic to hypogeic (and vice versa) occurred. The palaeogeographical evolution of European land, freshwater, and brackish water may have followed models like the Glacial-Sensitive Model (GSM) and ‘Sea-Level Sensitive’ dynamic model (SLS), which are available for use in marine island biogeography [42], thus shaping the speciation process.

## 7. Conclusions

The natural history and phylogenetic systematics of *Dendrocoelum* s.l. should be reinvestigated. An integrative approach combining paleobiogeography, morphology, biological features, and genetics may lead to a better understanding of this group and may lead to unexpected findings.

## 8. Future Directions of Study

Specific future research needs and questions

A reinvestigation of the Romanian Dendrocoelidae should take different approaches and should aim to answer the following questions:

1. The status of the described species. Some species may be synonymized, and new species may be described.
2. The genus or subgenus level of *Paradendrocoelum* and *Palaeodendrocoelum*. In this regard, the species *Paradendrocoelum alexandrinae* and *Palaeodendrocoelum getticum* are of particular interest.
3. The anophthalmic species *Palaeodendrocoelum getticum*, *Polycladodes voinovi,* and *Polycladodes affine* may support the validation of one of the hypotheses regarding their origin and paleogeographic distribution (the migration route). They can also help clarify eye character, whether it is homologous character (divergent evolution) or analogous (convergent evolution).
4. Molecular support for the timing of subterranean colonization. This can be used to validate eye/flagellum evolution hypotheses.

The Dendrocoelidae as freshwater flatworms in the era of genetics

Numerous modern genetic techniques, tools, and protocols provide new and deeper insights regarding freshwater flatworms. This group of worms is characterized by the presence of neoblasts throughout their lives. Neoblasts are a population of adult pluripotent stem cells involved in many processes, such as regeneration, turnover of specialized cells, and responses to external insults [43]. Stem cell research has focused on planarians (Dugesiidae), which became a model system due to their high regenerative abilities. The literature in this field is impressive; numerous research articles and reviews have revealed complex regulatory genetic networks in stem cell systems involved in development, tissue and organ function, and the repair of various parts of a worm [43,44,45,46,47,48,49].

Dendrocoelidae must have neoblasts and a stem cell system, which may have been lost or degraded during evolution, but, so far, this area remains largely unexplored. Only a few papers address regeneration in *Dendrocoelum lacteum* alone [50] or in other summative and comparative contexts [49].

Two main topics seem to be important for *Dendrocoelides’* geological evolution: neoblasts and eye loss. Most *Dendrocoelides* species found in the actual fauna are endemic and cavernicolous. It has been well-documented that eye loss comes as an adaptation to dark environments in many invertebrate and vertebrate phyla, including planarians [51,52,53]. Regarding the eyes, the following questions should be answered: How will a cavernicolous anophthalmic species react when exposed to light for a longer period? Will it develop eyes? What is the meaning of the acquisition or loss of eyes in this group of worms characterized by the presence of neoblasts?

In recent years, there has been increasing awareness of the role of the environment in producing phenotypes. An inherited genome can respond not only to mutations to produce new phenotypes but also to numerous environmental factors involving epigenetic control (for instance, DNA methylation) [54].

Other modern methodologies, such as eDNA [55], genome skimming [56,57,58], and microCT imaging [59,60,61], could yield new insights and outcomes in the study of these worms, as they have in other living beings, for various purposes and in different contexts.

## Figures and Tables

**Table 1:** Species inventory. The mark ? means unknown.

Species Name (System: Gourbault 1972 and Sluys et al., 2009)	Original Genus/Subgenus (in Primary Reference)	Type Locality/Loc. in Romania	Primary References (Species Original Description)	Literature Source (Used by Author)	Collection Holding Type Specimens
1. *D. (Dendrocoelides) sphaerophallus* (de Beauchamp, 1929)	*Dendrocoelides*	Pui (Hunedoara, Romania)	Beauchamp 1929	Beauchamp 1929 [12]	?
2. *D. (Dendrocoelides) chappuisi* de Beauchamp, 1932	*Dendrocoelum (Dendrocoelides ?)*	Babadag, Tulcea, Romania	Beauchamp 1932	Beauchamp 1932 [13]	?
3. *D. (Dendrocoelides) clujanum* Codreanu, 1943	*Dendrocoelum (Dendrocoelides)*	Cluj, Romania	Codreanu 1943	Codreanu 1943 [16]	?
4. *D. (Dendrocoelides) racovitzai* de Beauchamp, 1949	*Dendrocoelum (Dendrocoelides)*	Cloșani, Romania	Beauchamp 1949	Beauchamp 1949 [14]	?
5. *D. (Dendrocoelides) banaticum* Codreanu & Balcesco, 1967	*Dendrocoelum (Dendrocoelides)*	Oravița, Romania	Codreanu and Balcesco, 1967a	Codreanu and Balcesco, 1967a, b [18,19]	?
6. *D. (Dendrocoelides) atriostrictum* Codreanu & Balcesco, 1967	*Dendrocoelum (Dendrocoelides)*	Reșița, Semenic Mt., Romania	Codreanu and Balcesco, 1967a	Codreanu and Balcesco, 1967a, b [18,19]	?
7. *D. (Dendrocoelides) debeauchampianum* Codreanu & Balcesco, 1967	*Dendrocoelum (Dendrocoelides)*	Orșova, Romania	Codreanu and Balcesco, 1967a	Codreanu and Balcesco, 1967a, b [18,19]	?
8. *D. (Dendrocoelides) tismanae* Codreanu & Balcesco, 1967	*Dendrocoelum (Dendrocoelides)*	Tismana, Romania	Codreanu and Balcesco, 1967b	Codreanu and Balcesco, 1967b [19]	?
9 *D. (Dendrocoelides) stenophallus* Codreanu & Balcesco, 1967	*Dendrocoelum (Dendrocoelides)*	Sohodol, Romania	Codreanu and Balcesco, 1967b	Codreanu and Balcesco, 1967b [19]	?
10. *D. (Dendrocoelides) orghidani* Codreanu & Balcesco, 1967	*Dendrocoelum (Dendrocoelides)*	Lipova—Poiana Ruscă Mt., Romania	Codreanu and Balcesco, 1967c	Codreanu and Balcesco, 1967c [20]	?
11. *D. (Dendrocoelides) polymorphum* Codreanu & Balcesco, 1967	*Dendrocoelum (Dendrocoelides)*	Agigea, Romania	Codreanu and Balcesco, 1967c	Codreanu and Balcesco, 1967c [20]	?
12. *D. (?Paradendreocoelum?) alexandrinae* Codreanu & Balcesco, 1970,	*Dendrocoelum (Paradendrocoelum)*	Vama Buzăului, Romania	Codreanu and Balcesco, 1970	Codreanu and Balcesco, 1970 [21]	?
13. *D. (Apodendrocoelum) brachyphallus* (de Beauchamp, 1929)	*Dendrocoelides*	Vașcău, Romania	Beauchamp 1929	Beauchamp 1929 [12]	?
14. *D. (Apodendrocoelum) lipohallus* (de Beauchamp, 1929)	*Dendrocoelides*	Turda, Romania	Beauchamp 1929	Beauchamp 1929 [12]	?
15. *D. (Palaeodendrocoelum) romanodanubialis* (Codreanu, 1949–1950),	*Palaeodendrocoelum*	Iron Gates on Danube, Romania	Codreanu 1950	Codreanu 1950 [17]	?
16. *D. (Palaeodendrocoelum) getticum* Codreanu & Balcesco, 1970	*Dendrocoelum (Palaeodendrocoelum)*	Bucharest, Romania	Codreanu and Balcesco, 1970	Codreanu and Balcesco, 1970 [21]	?
17. *D. (Eudendrocoelum) botosaneanui* del Papa, 1965	*Dendrocoelum (Eudendrocoelum)*	Anina, Romania	Del Papa 1965	Gourbault 1972 [2]	?
18. *Polycladodes album* Steinmann, 1910,	*Polycladodes*	? Dobrogea, Romania	Steinmann 1910	Gourbault 1972 [2]	?
19. *Polycladodes voinovi*, Codreanu, 1929	*Polycladodes*	Sinaia, Romania	Codreanu 1929	Codreanu 1929 [15]	?
20. *Polycladodes affine* (Codreanu & Balcesco, 1970),	*Dendrocoelum (Polycladodes)*	Unspecified in South Făgăraș Mt., Romania	Codreanu and Balcesco, 1970	Codreanu and Balcesco, 1970 [21]	?
21. *Dendrocoelum obstinatum* Stocchino & Sluys, 2017, Romania, Dobrogea	*Dendrocoelum*	Movile Cave, Dobrogea, Romania	Stocchino et al. 2017	Stocchino et al. 2017 [3]	Naturalis Biodiversity Center, University of Sassari

## Data Availability

This paper is a review. The first submitted manuscript was not posted on a preprint server. No new data were analyzed in this study. Data sharing is not applicable to this article.
